# Assessing the association between vitamin D receptor and dental age variability

**DOI:** 10.1007/s00784-021-04140-y

**Published:** 2021-08-31

**Authors:** Erika Calvano Küchler, Julia Carelli, Nathaly D. Morais, João Armando Brancher, Celia Maria Condeixa de França Lopes, Flares Baratto-Filho, Eva Paddenberg, Maria Angélica Hueb de Menezes Oliveira, Alexandre Moro, Christian Kirschneck

**Affiliations:** 1 Department of Orthodontics, University of Regensburg, Franz-Josef-Strauss-Allee 11, 93053 Regensburg, Germany; 2 School of Health Science, Positivo University, Curitiba, Brazil; 3 Department of Dentistry, University of the Region of Joinville - Univille, Joinville, Brazil; 4 Center for Health and Biological Sciences, Pontifícia Universidade Católica do Paraná (PUCPR), Curitiba, Brazil; 5 School of Dentistry, University de Uberaba, Uberaba, Brazil; 6 Department of Orthodontics, Federal University of Paraná, Curitiba, Paraná, Brazil

**Keywords:** Vitamin D, Genetic polymorphisms, VDR, Dental maturity, Dental development

## Abstract

**Objectives:**

To explore the association between genetic polymorphisms in *vitamin D receptor* (*VDR*), vitamin D serum levels, and variability in dental age.

**Material and methods:**

This cross-sectional study was based on an oral examination, panoramic radiograph analysis, and genotype analysis from biological samples. Dental age was evaluated using two different methods: Demirjian et al. (Hum Biol 45:211–227, 1973) and Hofmann et al. (J Orofac Orthop.78:97–111, 2017). The genetic polymorphisms BglI (rs739837) and FokI (rs2228570) in *VDR* were genotyped through real-time PCR. The vitamin D level was also measured in the serum. Delta (dental age–chronological age) was compared among genotypes in *VDR* in the co-dominant model. Multiple linear regression analysis was also performed. An established alpha of 5% was used.

**Results:**

Genotype distributions of BglI and FokI were not associated with dental maturity (*p* > 0.05). In the logistic regression analyses, genotypes in BglI and FokI and vitamin D levels were not associated with variability in dental age (*p* > 0.05).

**Conclusions:**

The genetic polymorphisms BglI and FokI in *VDR* and the vitamin D levels were not associated with variability in dental age.

**Clinical relevance:**

To unravel the factors involved in dental maturity can improve dental treatment planning in pediatric and orthodontic practice.

**Supplementary Information:**

The online version contains supplementary material available at 10.1007/s00784-021-04140-y.

## Introduction

Dental development is a complex multilevel, multidimensional, and long progressive process. Multifactorial interactions involving genetic, epigenetic, hormonal, and environmental factors play a crucial role [1]. The development of permanent teeth spans from childhood to early adulthood with the maturation of the root apices of the third molars [2–11]. Dental development is a useful indicator of maturation in clinical practice, an estimator of age for minors, forensic identification, and archeological studies. In 1973, Demirjian et al. [2] introduced a method that estimates dental age based on the development of seven teeth from the lower left side of the mandible, scoring their calcification stages from A to H. In 2017, Demirjian’s original method of scoring was adopted by Hofmann et al. [11] for age assessment based on third molar maturity.

Vitamin D is a secosteroid hormone, which plays an important role in calcium homeostasis and is vital for tissue mineralization [12]. The biological effects of vitamin D are mediated by binding to its intracellular receptor, called the vitamin D receptor (VDR), a member of the nuclear receptor superfamily [13]. VDR is essential in the mediation of mineral metabolism and the control of calcium and phosphate metabolism. The gene encoding the VDR in humans is located on chromosome 12 [14] and has many polymorphic regions [15].

There is some evidence from studies with animal models and humans demonstrating that both vitamin D and VDR are involved in dental development, including enamel and dentine mineralization [16–19]. Therefore, our hypothesis is that the gene encoding VDR is involved in individual variability of dental age. Thus, in this study, we used the eight-stage method of Demirjian et al. [2] to explore the association between genetic polymorphisms in *VDR*, vitamin D status, and variability in dental age.

## Materials and methods

This project was approved by the Local Ethical Committee (3.036.106) and was conducted in accordance with the Declaration of Helsinki. Informed consent and assent forms were signed by all legal guardians and patients. This study was conducted following the Strengthening the Reporting of Genetic Association study (STREGA) statement checklist (supplementary material).

This cross-sectional study consisted of a consecutive sample of children, selected from patients seeking orthodontic treatment at the Positivo University (Curitiba, Brazil) from 2018 to 2019. An analysis comprising anamnesis, oral examination, panoramic radiographs, and biological sample collection was performed for each patient and all patients were posteriorly enrolled in orthodontic treatment.

We included patients of both genders with the age ranging from 10 to 16 years. Children with systemic conditions, syndromes, oral clefting, bone disease, or history of any serious trauma or injury of the face, as well as those who had previously undergone orthodontic treatment, were excluded.

All individuals were submitted to cone-beam computed tomography (CBCT) before orthodontic treatment as part of routine orthodontic diagnostics for treatment planning. CBCT scans were performed following a standardized protocol in habitual occlusion with head alignment according to the Frankfort horizontal plane, a scanning time of 17.8 s, a field of view of 170 mm/170 mm, and an exposure of 120 kVp/8 mA with an i-CAT (Imaging Sciences International, Hatfield, PA, USA), model 9140. The CBCT images were exported as DICOM (Digital Imaging and Communication in Medicine) files with a voxel size of 0.3 mm. Panoramic images were reconstructed from CBCT volumes.

## Chronological age and dental age evaluation

The chronological age in years (two decimals) was calculated for each child by subtracting the date of birth from the date of the imaging exam and blinded during the evaluation of dental age. Intrarater and interrater reliabilities were examined by using weighted kappa statistics. Five randomly selected participants were evaluated twice in a blinded manner both by the same investigator as well as by a second (senior) investigator.

Dental age was estimated using two different methods [2, 11]. The dental age estimation according to Demirjian is based on the calcification status of seven permanent teeth at the left side of the mandible (except the third molar) defining 8 different developmental mineralization stages “A” to “H” for each tooth starting with the initial crown formation and ending with the closure of the root apex. Considering the mineralization stage of each tooth, a score can be derived from the table provided by Demirjian et al. [2] and converted into the corresponding dental age.

The Hofmann method is a simplified version of the Demirjian method based on third molar mineralization aimed to extend the age range of applicability to higher ages [11]. Briefly, for each third molar, one of the same eight developmental mineralization stages “A” to “H” according to Demirjian et al. [2] was defined and matched with a jaw- and gender-specific point score, which can be translated to a corresponding dental age as described by Hofmann et al. [11].

To allow further comparison between dental ages, a delta for each child was calculated by subtracting his or her chronological age (CA) from the dental age (DA): delta = DA − CA. The delta was calculated for both methods.

### Quantification of serum Vitamin D levels

Serum vitamin D levels were measured by chemiluminescence microparticle immunoassay with an Abbott Alinity automated immunoassay analyzer (Abbott Laboratories, IL, USA) that uses an anti-analyte coated with paramagnetic microparticles and anti-analyte acridinium-labeled conjugates [20]. The reactions were performed according to the manufacturer’s instructions. Alinity-Abbott calibrators were used to adjust the equipment for the analytical measurement range for vitamin D and the results are given in nanograms per milliliters. Less than 20 ng/mL (50 nmol/L) were defined as vitamin D deficiency, 21 to 29 ng/mL (51–74 nmol/L) as vitamin D insufficiency, and 30 to 100 ng/mL (75–250 nmol/L) as vitamin D sufficiency [21].

### Genomic DNA extraction and allelic discrimination analysis of VDR

Saliva samples were also collected from each child for the extraction of genomic DNA from buccal epithelial cells. DNA extraction followed an established, previously published protocol [22]. DNA concentration and purity were determined by spectrophotometry using a NanoDrop 1000 (Thermo Scientific Inc., Waltham, MA, USA).

Allelic discrimination reactions (genotyping) were performed with real-time polymerase chain reactions (PCR), TaqMan technology (Applied Biosystems®, StepOnePlus Real-Time PCR System, Thermo Fisher Scientific, Foster City, CA, USA), to evaluate two genetic polymorphisms in *VDR*: a UTR variant called BglI (rs739837, G > T) and a missense variant called FokI (rs2228570, A > G/ Met > Thr).

### Statistical analysis

GraphPad Prism 8.2 (GraphPad, San Diego, CA, USA) was used. Shapiro–Wilk tests were used to test the normality of the data. One-way ANOVA with Tukey’s post hoc tests and Kruskal–Wallis *H* tests with Dunn’s post hoc tests were used for comparisons of means and standard deviations (SD) of differences (delta) of dental age (DA-CA) among genotypes in *VDR* in the co-dominant model. Multiple linear regression analysis was performed using the genotypes in the co-dominant model, gender (male and female), and vitamin D serum levels. Cases with missing values were dropped from the corresponding analysis. The chi-square test was used to assess the Hardy–Weinberg equilibrium. Significance was assumed at *p* < 0.05.

## Results

Of the initial 37 patients screened, complete clinical and biological data of 36 individuals were available for analysis (Supplementary Fig. 1).

Sample characteristics are presented in Table 1. Serum vitamin D levels ranged from 10.5 to 51.5 (mean = 23.5, *SD* = 1.45).

**Table 1:** Sample characteristics

Gender, *n* (%)	
Male	17 (47.2%)
Female	19 (52.8%)
Age group in months, *n* (%)	
120 to 156 months old	19 (52.8%)
157 to 192 months old	17 (47.2%)
Chronological age in years	
Minimum–maximum	10–16
Mean (standard deviation)	12.8 (SD 1.7)
Dental maturation according to the Demirjian method (years)	
Minimum–maximum	9.4–17
Mean (standard deviation)	13.8 (SD 1.7)
Delta DA-CA (years) for Demirjian’s method	
Minimum–maximum	− 1.48–5.2
Mean (standard deviation)	0.99 (SD 1.46)
Dental maturation according to the Hofmann method (third molars) (years)	
Minimum–maximum	10.5–18.8
Mean (standard deviation)	14.1 (SD 2.1)
Delta DA-CA (years) for Hofmann’s method	
Minimum–maximum	− 2.69–6.63
Mean (standard deviation)	1.42 (SD 1.99)
Vitamin D status, *n* (%)	
Deficient	15 (41.7%)
Insufficient	12 (33.3%)
Sufficient	9 (25.0%)

**Fig. 1:**
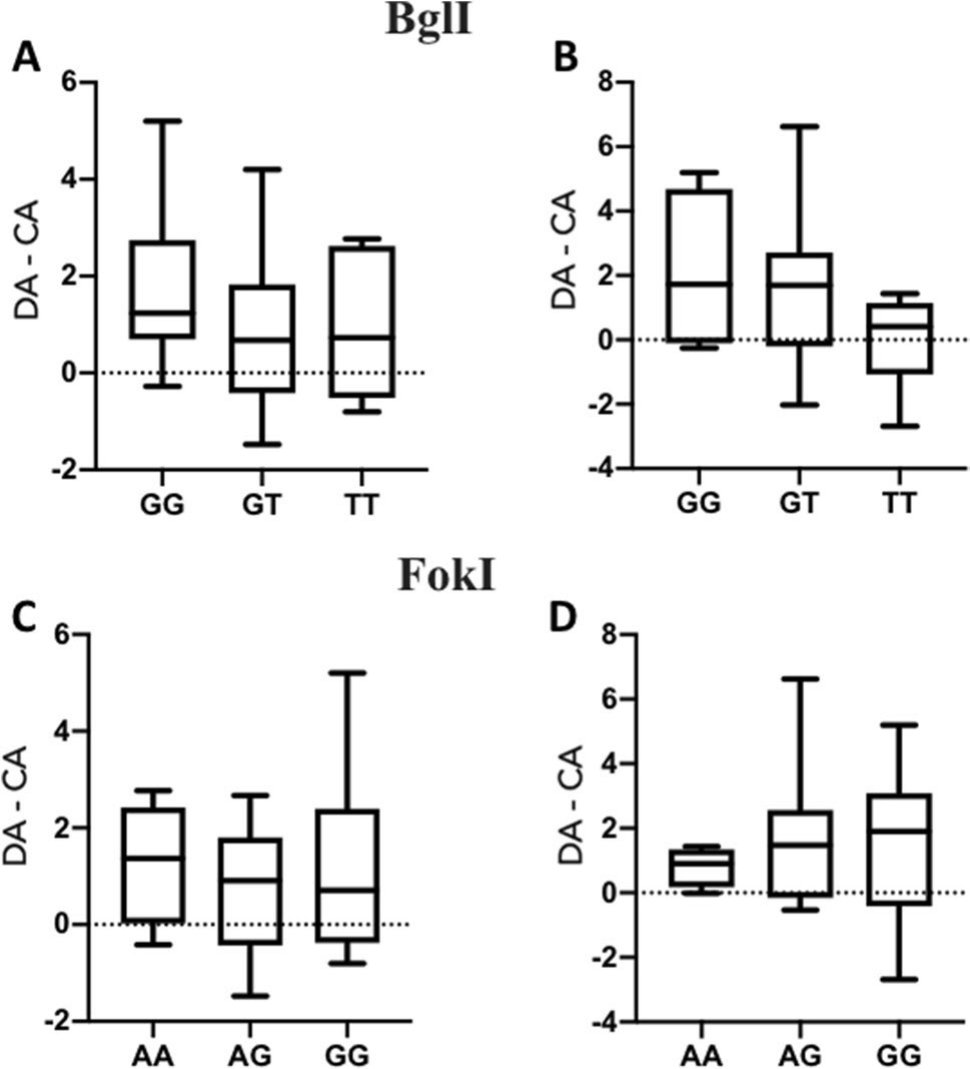
Dental age variability (difference of dental and chronological age DA-CA) according to *VDR* genotypes. **A** Dental age (according to Demirjian’s method) distribution according to the genotypes in BglI. **B** Dental maturity (according to Hofmann’s method) distribution according to the genotypes in BglI. **C** Dental maturity (according to Hofmann’s method) distribution according to the genotypes in FokI. **D** Dental maturity (according to Hofmann’s) distribution according to the genotypes in FokI. DA means dental age; CA means chronological age

In genetic polymorphism BglI, 6 patients presented the GG genotype, 20 the GT genotype, and 6 patients the TT genotype (Hardy-Weinberg_Chi-square_ = 2.00). In genetic polymorphism FokI, 4 patients had the AA genotype, 12 patients the AG genotype, and 16 patients the GG genotype (Hardy-Weinberg_Chi-square_ = 0.51). Figure 1 shows the differences in dental and chronological age (DA-CA) for both methods according to the genotypes in BglI and FokI. Genotype distributions were not associated with variability in dental age: BglI (*p* = 0.584) and FokI (*p* = 0.782) for dental age according to Demirjian et al. and BglI (*p* = 0.220) and FokI (*p* = 0.823) according to Hofmann et al., respectively.

Table 2 presents the results of the multiple regression analysis. Genotypes in BgII and FokI and vitamin D serum had a weak, not statistically significant effect on the variability of dental age.

**Table 2:** Multiple linear regression analysis

Phenotype	Variable	Beta	SE	95% CI		*t*	*p*-value
				Upper	Lower		
Demirjian	BglI (GT)	0.216	0.904	− 1.646	2.078	0.238	0.813
	BglI (GG)	1.202	1.128	− 1.121	3.524	1.066	0.296
	FokI (AG)	− 0.337	0.656	− 1.691	1.015	0.514	0.611
	FokI (AA)	0.525	1.108	− 1.756	2.808	0.474	0.639
	Vitamin D levels	0.006	0.035	− 0.066	0.078	0.173	0.863
Hofmann	BglI (GT)	1.941	1.227	− 0.603	4.486	1.582	0.127
	BglI (GG)	2.715	1.519	− 0.435	5.867	1.787	0.087
	FokI (AG)	0.031	0.951	− 1.942	2.006	0.033	0.973
	FokI (AA)	0.472	1.495	− 2.627	3.572	0.316	0.754
	Vitamin D levels	− 0.014	0.047	− 0.114	0.084	0.310	0.758

SE means standard error. CI means confidence interval. For BgII, the reference was the TT genotype. For FokI, the reference was GG genotype

## Discussion

The high prevalence of vitamin D deficiency is worrisome and may impact health, especially during childhood and pregnancy. A recent systematic review identified 195 studies conducted in forty-four countries involving more than 168,000 participants. The authors reported that mean serum levels of vitamin D varied considerably across studies, with 37.3% of the included studies reporting mean levels below 50 nmol/L [23]. In our study, we also observed a low mean vitamin D serum level, corresponding to 41.7% of children being vitamin D deficient. In general, the major cause of vitamin D deficiency is considered to be a lack of sunlight exposure with inadequate exposure to solar ultraviolet B rays [23, 24]. Children included in this study live in Curitiba, a city located in the south of Brazil in the latitude 25°S, and investigations were performed during winter. However, studies investigating vitamin D status in Brazil conducted over the past 10 years demonstrated a high prevalence of vitamin D insufficiency in Brazil in different latitudes across the country, even in some regions closer to the equator [25].

Although in our sample vitamin D level was not associated with dental age variability, studies with vitamin D-deficient mice suggested that vitamin D deficiency impacts tooth development [16]. It is important to highlight that our results should be interpreted with caution. Dental development is a continuous process and vitamin D level was tested only at one particular time point in our patients. Longitudinal studies could aid in elucidating the impact of vitamin D serum levels on the variability of dental development.

Animal and human studies also suggested that VDR plays an important role in dental development and could affect dental maturity. In hypo-calcemic null mutant *vdr*(− / −) mice, dento-alveolar bone was hypomineralized [19]. A study evaluating dental maturity in children with hereditary vitamin D-resistant rickets (a rare genetic disorder caused by mutations in *VDR*) reported that dental development represents an indicator of the disease progressing, initially protected by maternal blood levels of calcium and later restored by therapeutic supplies that normalize these levels [26]. The cellular actions of vitamin D are mediated via the VDR that modulates and regulates the expression of many genes (estimated 5–10% of the entire genome) [27]. The genetic polymorphism BglI (rs739837) is located near the stop codon in exon 9, while the genetic polymorphism FokI (rs2228570) results in frameshift mutations and introduces a premature methionine start codon resulting in different VDR protein structures and functions [28]. Both polymorphisms were previously associated with a variety of conditions (NCBI). In our study, BglI and FokI were not associated with dental age variability. However, it is possible that the sample size per genotype could in part have yielded falsely negative results. Future studies should continue evaluating the association between dental age variability and polymorphisms in *VDR* in a larger sample.

In the literature, there is a range of classifications for evaluating dental age. Such classifications were presented by Gleiser and Hunt in 1955 [4], Nolla in 1960 [5], Moorrees et al. in 1963 [6], Demirjian et al. [2] in 1973, Haavikko’s in 1974 [7], Coutinho et al. [3] in 1993, Kullman in 1995 [8], Willems in 2001 [9], London Atlas in 2014 [10], and Hofmann et al. in 2017 [11]. Some of these methods identify a large number of stages that are difficult to delimit from one another. On the other hand, Demirjian’s method differentiates only four stages of crown development (stages A to D) and four stages of root development (stages E to H). All the stages are easily defined by changes in morphology. In a previous study, Dhanjal et al. [29] concluded that the Demirjian method performed best for intra- and interexaminer agreement and also for the correlation between chronological and dental age. Therefore, Demirjian’s method was selected for our study.

It is important to highlight that Hofmann’s method also used Demirjian’s method to classify dental age according to the development of third molars to extend the age range to early adulthood. We decided to include Hofmann’s method, as some of our included patients’ age ranged from 14 to 16 years. Age estimation using tooth development becomes difficult after 14 years of age since all permanent teeth except the third molars would have completed their dental development and calcification [30].

In our study, we decided to evaluate only dental development stages, as methods evaluating dental eruption are influenced by various factors such as tooth extractions, ankylosis, ectopic positions, and persistence of primary teeth. Dental development is assumed to be a more reliable criterion for determining dental age than tooth eruption [5].

Briefly, in past years, vitamin D has been gaining growing attention also in the fields of oral health and dental alterations [24]; however, to the best of our knowledge, this is the first study to explore the association between *VDR*, vitamin D, and dental age variability.

## Conclusion

This is the first study to explore the association between VDR, vitamin D, and dental age variability. The genetic polymorphisms BglI (rs739837) and FokI (rs2228570) in *VDR* and vitamin D serum levels were not associated with dental age variability.

## Supplementary information

Below is the link to the electronic supplementary material.

Supplementary file1 (PNG 139 KB)
