# Supplementary figures and images for: Assessing the association between vitamin D receptor and dental age variability
Source: Clin Oral Investig. 2021 Aug 31;26(2):1677–82. doi: 10.1007/s00784-021-04140-y (PMC8816742; doi:10.1007/s00784-021-04140-y)

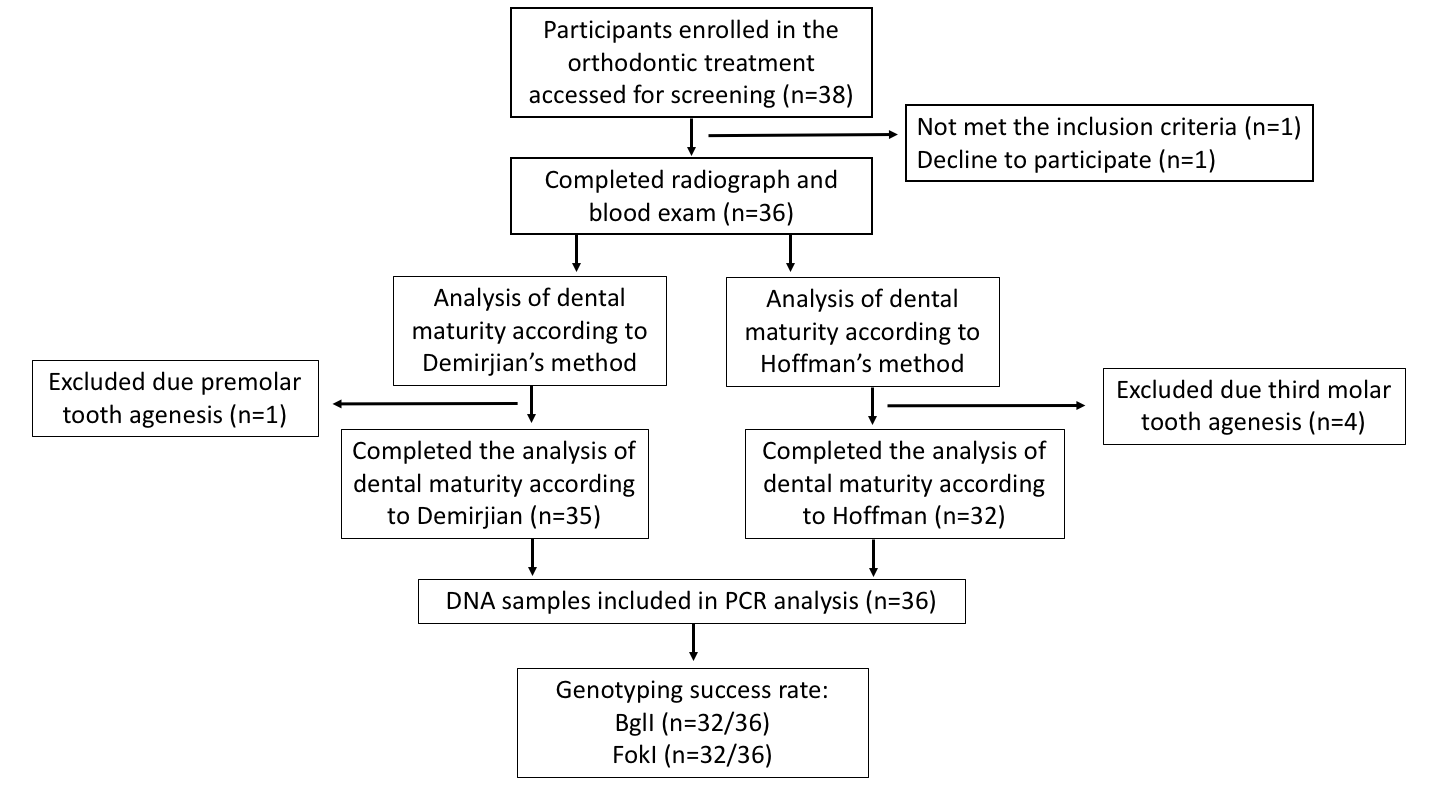

Supplement: Supplementary file 1 — Supplementary file1 (PNG 139 KB) [file 784_2021_4140_MOESM1_ESM.png]
